# Peripheral cortisol administration blunts reward arousal but heightens anxiety-like arousal in marmosets

**DOI:** 10.1192/bjo.2021.453

**Published:** 2021-06-18

**Authors:** Laith Alexander, Philip Gaskin, Lauren McIver, Angela Roberts

**Affiliations:** 1 St Thomas' Hospital, University of Cambridge; 2 University of Cambridge

## Abstract

**Aims:**

Excess hypothalamic-pituitary-adrenal (HPA) axis activation is common in people with major depression and generalised anxiety disorder. We sought to determine whether higher circulating levels of the glucocorticoid cortisol are causally related to the expression of anhedonia-like and anxiety-like behaviours in marmosets.

**Method:**

Four marmosets (two male, two female) took part in the study. Cortisol and saline control injections were administered intramuscularly and salivary cortisol samples were taken before and after injections to determine if circulating cortisol levels changed from pre- to post-injection. To measure anhedonia-like behaviours, we trained marmosets on an appetitive Pavlovian conditioning paradigm, where animals learn to associate two anticipatory auditory cues (conditioned stimulus + or conditioned sitmulus -, CS+ or CS-) with the presence or absence of food reward (unconditioned stimulus + or unconditioned stimulus -, US+ or US-). Using cardiovascular telemetry probes and video cameras, we recorded animals' cardiovascular and behavioural arousal in freely moving conditions, comparing the injection of saline control versus 5mg/kg, 10mg/kg or 20mg/kg intramuscular cortisol. To measure anxiety-like behaviours, we used a human intruder (HI) paradigm, where marmosets are confronted with an unfamiliar human in their home cage. We recorded their behaviour on video cameras after saline control or 20mg/kg intramuscular cortisol. We used an exploratory-factor analysis (EFA) to determine how marmosets' behaviours towards the intruder loaded onto an 'anxiety-like' score. We then compared these scores under saline control versus cortisol conditions. Significance was set at p < 0.05.

**Result:**

Unlike saline control, we found that subcutaneous injections of 20 mg/kg cortisol successfully elevated peripheral cortisol concentrations to levels equivalent to peak circadian concentrations (p = 0.023). In the appetitive setting, 5 mg/kg, 10 mg/kg and 20 mg/kg cortisol injections blunted anticipatory (CS+ induced) increases in behavioural arousal (p = 0.004) but did not alter anticipatory cardiovascular arousal. Consummatory behavioural and cardiovascular arousal also remained intact. In the HI test, 20 mg/kg cortisol injections moderately increased anxiety towards the intruder as measured by an increase in marmosets' EFA-derived anxiety-like scores (p = 0.035).

**Conclusion:**

In marmosets, elevated peripheral cortisol levels are causally related to the behavioural features of blunted reward anticipation together with elevated anxiety-like behaviours characteristic of mood and anxiety disorders. Future work will characterise the neuroimaging changes induced by elevated peripheral cortisol levels and identify the regions of the prefrontal cortex contributing to HPA axis regulation and dysregulation.

